# The impact of hyperglycaemia and/or type 2 diabetes on women with breast cancer undergoing or post-cytotoxic chemotherapy: a systematic literature review

**DOI:** 10.1007/s00520-026-10947-w

**Published:** 2026-06-30

**Authors:** Anne Crockett, C. McCaughey, M. Dornan, M. Donovan, E. Aughey

**Affiliations:** 1 School of Nursing and Midwifery, Queen’s University Belfast, Belfast, Northern Ireland; 2 Belfast Health and Social Care Trust, Belfast, Northern Ireland

**Keywords:** Breast neoplasm, Chemotherapy, Hyperglycaemia, And diabetes

## Abstract

**Purpose:**

Hyperglycaemia and/or type 2 diabetes (T2D) can have a detrimental effect on women with breast cancer (BC) undergoing or post-cytotoxic chemotherapy. This systematic review aims to evaluate the short and long-term consequences of hyperglycaemia and/or T2D on treatment outcomes in women with breast cancer receiving cytotoxic chemotherapy.

**Methods:**

Studies published between 2018 and 2024 across four electronic databases were identified. The JBI critical appraisal tool was adopted to select high-quality studies.

**Results:**

Nine papers met the criteria for review. Thematic analysis identified two themes: 1) short-term consequences of hyperglycaemia and/or T2D, specifically its impact on healthcare utilisation, treatment toxicity, and treatment modification, and 2) long-term consequences of hyperglycaemia and/or T2D, such as effects on pathological response, prognosis, and mortality.

**Conclusion:**

Proactive identification and rigorous management of hyperglycaemia and/or T2D are essential to reducing complications and improving outcomes in women with BC receiving chemotherapy. Evidence demonstrates that poor glycaemic control clearly impairs treatment response. The current research gap and fragmented care pathways demand strengthened multidisciplinary collaboration and the delivery of personalised care. These measures are necessary to significantly improve the quality of living with and beyond a diagnosis of BC.

**Supplementary Information:**

The online version contains supplementary material available at 10.1007/s00520-026-10947-w.

## Introduction

Breast Cancer (BC) remains the most prevalent cancer among women globally [1–3]. It is a multifactorial disease influenced by factors such as age, sex, obesity, lifestyle, and diet [4–6]. While more than 90% of cases are nonmetastatic at diagnosis [7], treatment strategies are highly individualised, considering tumour characteristics, disease stage, patient health, and molecular profiles [8]. Despite rising incidence rates, mortality has declined due to advancements in treatment [5, 9]. Multimodality treatment encompasses surgery, radiotherapy, hormonal therapy, and systemic anticancer therapies (SACT), including chemotherapy [10, 11]. Combination and sequential anthracycline and taxane-based chemotherapy regimens are the gold standard for early BC, reducing BC mortality by one-third [5, 12]. However, type 2 diabetes (T2D) and hyperglycaemia can negatively impact chemotherapy efficacy [8, 13]. This is a growing concern given the increasing prevalence of comorbidities such as cardiovascular disease, obesity, and T2D in the ageing population [14, 15].

It is estimated that 20% of individuals with cancer have co-occurring T2D at diagnosis [16], with T2D being the most prevalent form [17]. Characterised by diminished insulin secretion and heightened insulin resistance [17], T2D may develop secondary to medications or underlying illnesses. Various cytotoxic chemotherapies and supportive treatments can worsen T2D progression [13, 18]. For example, corticosteroid therapy can cause steroid-induced hyperglycaemia (SIH), which affects approximately 32% of steroid-treated individuals [17, 19]. Consequently, random blood glucose (RBG) monitoring is essential for identifying hyperglycaemic episodes during treatment. Together, these factors create challenges in women with BC undergoing chemotherapy, compromising glycaemic control and potentially undermining treatment outcomes. For this reason, this review will focus on the impact of T2D and SIH in women with breast cancer receiving cytotoxic chemotherapy.

A concurrent diagnosis of T2D and cancer is increasingly common, with a complex interplay between the two conditions [18–21]. The risk of chemotherapy-induced T2D varies by drug class, ranging from up to 10% with anthracyclines (e.g., doxorubicin) to 66–67% with alkylating agents (e.g., busulfan) [21]. Additionally, corticosteroids such as dexamethasone, which are commonly used to mitigate chemotherapy-related toxicity, contribute to hyperglycaemia [22, 23]. However, guidance on the management of SIH is limited [18, 21].

Managing hyperglycaemia in women with BC undergoing chemotherapy remains a major clinical challenge, attempting to balance therapeutic benefits and adverse metabolic effects [8]. Cancer treatment is often prioritised over diabetes management [14], potentially leading to suboptimal care and poorer outcomes for individuals with both BC and T2D. Screening for T2D prior to chemotherapy is crucial, as recognised by the UK Chemotherapy Board [21], although this is not yet fully implemented in clinical practice. This gap in care, coupled with the recurring incidence of hyperglycaemia in oncology settings, highlights a critical opportunity to address a potential gap in knowledge and evidence-based practice. This systematic review aims to identify and synthesise evidence on the impact of hyperglycaemia and/or T2D in women with BC receiving cytotoxic chemotherapy, specifically examining both short- and long-term consequences for treatment outcomes and survival. This synthesis will inform evidence-based recommendations for clinical practice and identify priorities for future research.

## Methods

### Search strategy

Following Paré and Kitsiou’s framework [24], this systematic review followed six key steps: developing the research question, conducting the literature search, screening, data extraction, quality assessment, and data analysis. The PRISMA-S checklist guided the reporting of the search strategy [25], ensuring a rigorous and methodical approach.

### Formulating a research question

The well-established Population, Intervention, Context, and Outcome (PICO) framework guided the formulation of the research question, the literature search, study selection, and data analysis [26–28].

### Identification of key terms

A combination of key search terms, MeSH terms and appropriate use of truncation were applied to ensure that critical studies were identified (Table 1).

**Table 1:** Identified search terms

Search Terms using Boolean Operators				
**Concept 1** OR	**AND**	**Concept 2** **OR**	**AND**	**Concept 3** **OR**
Breast neoplasm		Chemotherapy*		Hyperglycaemia Hyperglyc#emia
Breast carcinoma				Glycaemic control Glyc#mic control
Breast Tumo#r				T2D Mellitus
Breast Cancer				

* #? = MeSH term

### Searching literature

Electronic searches were conducted across four academic databases — CINAHL, PsycINFO, Medline, and Web of Science — using three key search terms (Table 1) within a six-year timeframe (2018–2024). Three studies were identified through a Google Scholar search. Google Scholar can find over three-quarters of open-access publications alone [29]. To ensure a comprehensive literature search, Google Scholar is essential for gathering all relevant evidence. Citation chaining ensured a complete list of literature was obtained; however, no additional studies were found [30].

### Inclusion and exclusion criteria

To ensure greater objectivity and avoid bias or error, the inclusion/exclusion criteria were applied (Table 2) [24].

**Table 2:** Eligibility criteria for the search strategy

**Inclusion Criteria**
• Full-text article only
• Written in the English language only
• Published between the years 2018 to 2024
• Peer reviewed, empirical studies
• Participants over the age of 18
• Females diagnosed with Breast Cancer
• Participants receiving cytotoxic chemotherapy and who develop hyperglycaemia and/or T2D as the consequences of treatment
• Participants receiving cytotoxic chemotherapy and have preexisting T2D
**Exclusion Criteria**
• Participants that are receiving other anticancer therapies that do not include chemotherapy

All relevant studies were identified, and duplicates were removed using EndNote [25], leaving 86 abstracts for screening. Two reviewers (AC & CMcC) independently screened titles and abstracts, followed by full-text review. Nine studies were included in this systematic review (Table 3).

**Table 3:**
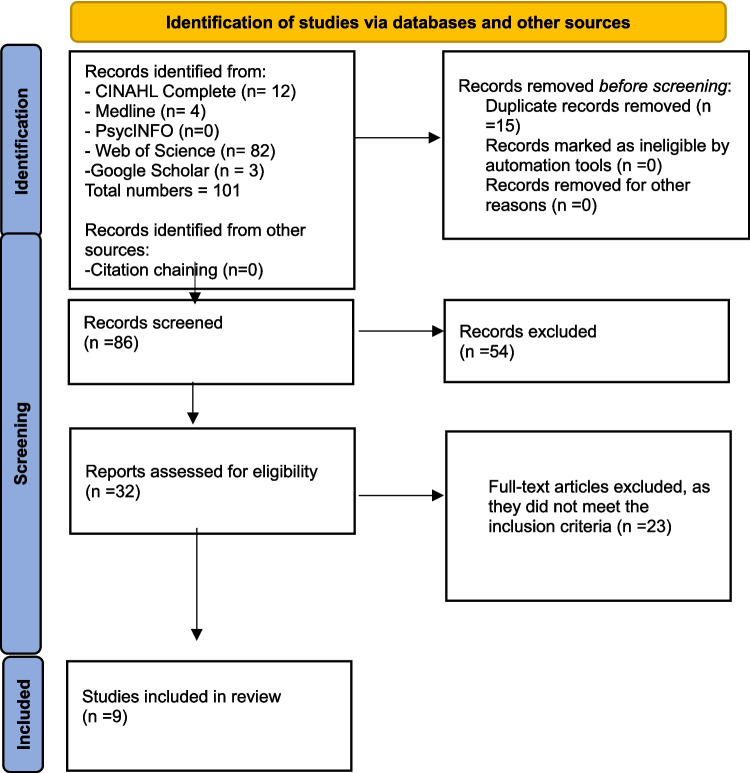
PRISMA 2020 flow diagram

### Data extraction/quality appraisal/data analysis

This systematic review employed the internationally recognised Joanna Briggs Institute (JBI) checklist [31] to assess the quality of all nine studies, which ranged from moderate to strong and were suitable for inclusion. All studies met the inclusion criteria, with quality assessed as strong, moderate, or weak according to the JBI criteria; the nine included studies were of moderate or strong quality. Common limitations included retrospective design and incomplete diabetes monitoring data.

Data extraction was performed independently by two reviewers (AC & CMC), with disagreements resolved through discussion or consultation with a third reviewer, EA, a BC Nurse Consultant. Thematic analysis was employed to identify recurring themes [32].

## Results

This review included nine studies published between 2018 and 2024 (Table 4). The included studies encompass diverse geographical locations, including North America, the Middle East, and South Asia, promoting the generalisability of the findings [33, 34]. All nine studies included only female participants diagnosed with invasive nonmetastatic BC (NMBC), also referred to as early BC or stage I-III BC. Multicentre studies were included (Table 4), enhancing the external validity and generalisability to broader populations [35, 36]. Five of the studies were single-centre (Table 4), potentially introducing bias and limiting generalisability [33].

**Table 4:** Characteristics of included studies

Authors and Date	Country of Origin	Sample Size	Design	T2D Confirmed (n/N)	Follow-up Period	Multicentre/Single Centre (Setting)	Data Obtained	Adjustment for Variables
Bekele et al. 2024	USA	3,704	Population-based retrospective cohort study	765/3,704	2007–2015 (8 years)	Population-based (statewide)	Linked Missouri Cancer Registry and Medicaid claims data	Age, race/ethnicity, socioeconomic status, cancer stage, breast cancer subtypes, surgery type
Javed et al. 2024	Pakistan	1,372	Retrospective cohort study	345/1,372	2016–2021 (5 years)	Single centre	Medical records	Age, BMI, smoking status, clinical stage, molecular subtype
Mahin et al. 2023	USA	100	Retrospective cohort study	67/100	2017–2019 (2 years)	Single centre	Medical records	Age, race, BMI, comorbidities and tumour characteristics
Phillips et al. 2023	USA	243	Case–control study	48/243	2019–2021 (2 years)	Single centre	Electronic health records	Age, BMI, cancer stage, smoking status, cardiovascular disease
Kleckner et al. 2022	USA	T = 674 BC = 439 C = 235	Retrospective cohort study	BC = 51/439 C = 16/235	2011–2013 (2 years)	Multicentre	Self-reported data and medical records	Age, BMI, exercise habits, smoking and other lifestyle habits
Ahn et al. 2020	Korea	423	Retrospective cohort study	82/423	2010–2015 (5 years)	Single centre	Medical records	Age, hypertension, BMI, surgical procedure etc
Arici et al. 2020	Turkey	135	Case–control study	25/135	2013–2017 (4 years)	Single centre	Medical files	T2D status, age, tumour characteristics and treatment status
Accordino et al. 2020	USA	4,373	Retrospective cohort study	1,082/4,373	2005–2013 (8 years)	Multicentre	SEER medical database	Age, race, SES, and receipt of chemotherapy, comorbidities such as hypertension
Lega et al. 2018	Canada	T = 14,865 BC = 4,955 C = 9,910	Retrospective cohort study	4,955/14,865	2007–2012 (5 years)	Multicentre	Linked population-based health databases	Age, socioeconomic status, tumour characteristics

*T* total sample, *C* comparator/control group (no breast cancer). T2D Confirmed (n/N) = number with confirmed T2D/total sample. Data source terminology (medical records, medical files, electronic health records) reflects the original language used in each study

The prevalence of diabetes across studies ranged from 19.75% [37] to 25.1% [38], reflecting the diverse population demographics and healthcare settings. The included studies demonstrated considerable methodological diversity. Nine studies employed retrospective designs: seven cohort studies [38–44] and two case–control studies [37, 41]. Sample sizes ranged from 100 [39] to 14,865 [42]. Follow-up periods varied from 2 years [37, 44] to 8 years [38, 43]. Diabetes was defined by medical history and medication use in six studies [37–40, 42, 44], while three incorporated biochemical criteria [41, 43, 45]. This heterogeneity necessitated a thematic rather than a meta-analytic synthesis.

Thematic analysis of study findings was conducted independently by two reviewers [32]. Initial codes were developed from the study outcomes and then grouped into themes through iterative discussions until consensus was achieved.

Two key themes were identified:

- Short-term consequences of hyperglycaemia and/or T2D encompass three subthemes: (i) healthcare utilisation, (ii) treatment-related toxicities, and (iii) treatment modifications; and 2) Long-term consequences of hyperglycaemia and/or T2D, comprising three subthemes: (i) pathological response, (ii) prognosis, and (iii) mortality outcomes.

### Short-term consequences

#### Healthcare utilisation

Compared with women with NMBC without T2D, those with T2D have more emergency department visits (52% vs. 33%), unplanned inpatient admissions (35% vs. 19%), and prolonged hospital stays [37]. Sustained SIH increases healthcare utilisation due to complications such as tissue damage, infections, and cardiovascular disease [39]. This issue is further complicated by delayed or undetected T2D diagnosis post-chemotherapy due to disjointed healthcare. One study highlighted the gap in primary care in managing women with new T2D post-BC diagnosis and chemotherapy, noting that while secondary care specialist visits increased substantially following BC treatment (median 19 to 46 per patient per year), primary care visits remained unchanged (median 2 per year for both groups) [40]. Increased visits to secondary care in women with a new diagnosis of T2D are evident; however, this is not reflected in primary care, which is a concerning result for vulnerable individuals who no longer receive secondary care for chemotherapy. These studies collectively emphasise the critical need for proactive glycaemic management in individuals with BC to mitigate complications, reduce hospitalisations, and improve overall treatment outcomes [37, 40].

#### Treatment toxicity

The need for better glycaemic control and surveillance of the development of hyperglycaemia and/or T2D is necessary to reduce treatment-related toxicities [37, 44]. Women with NMBC and T2D had a 54% greater incidence of infection than did women with NMBC without T2D (26%, *p* < 0.001). Furthermore, women with NMBC and T2D had significantly higher rates of hyperglycaemia (RBG > 180 mg/dL) during treatment compared to women with NMBC without T2D (96% vs. 40%, p < 0.001). Infection and neutropenic fever represented 46% of unplanned inpatient admissions for individuals with or without T2D. Additionally, women with NMBC without T2D who had hyperglycaemia (RBG > 180 mg/dL) during the study period had higher incidences of neutropenia, fever, and infection than women with BC without an RBG > 180 mg/dL [37]. T2D can also significantly impact fatigue in women with BC undergoing chemotherapy. One multicentre study [44] explored the association between T2D and cancer-related fatigue using the total Multidimensional Fatigue Symptom Inventory (MFSI) score across three time points: pre-chemotherapy, post-chemotherapy, and six months post-chemotherapy. Women with T2D had greater baseline fatigue (p = 0.017). Diabetes was associated with clinically meaningful worse fatigue throughout the study period among patients with BC (4.5 ± 2.0 points, *p* = 0.023) [44], highlighting the vital link between T2D and cancer-related fatigue.

#### Treatment modification

Women with NMBC and T2D faced significantly higher rates of chemotherapy dose omission than did NMBC women without T2D (23% vs. 9%) [37]. Additionally, women with diabetes with NMBC were unable to complete their planned regimen compared with NMBC women without T2D, retrospectively (19% vs. 6%) [37]. Furthermore, women with NMBC, regardless of their T2D status, who experienced hyperglycaemia (as indicated by RBG > 180 mg/dL) during the study period required treatment modifications (p < 0.05) [37], highlighting the persistent influence of glycaemic control on chemotherapy outcomes. The most common reasons for chemotherapy dose reduction were myelosuppression, neuropathy, and infection [37]. Treatment modifications, including dose omission and incomplete regimens, were reported more frequently in individuals with NMBC, regardless of their T2D status, who had hyperglycaemia (RBG > 180 mg/dL) during the study (*p* < 0.05). Additionally, women with NMBC with an HbA1c ≤ 7 had a greater incidence of not completing planned chemotherapy [37].

SIH impacts the number of chemotherapy cycles an individual can receive [39]. Women who remained euglycaemic (RBG < 140 mg/dL) received a median of 5 cycles of chemotherapy. In contrast, individuals with lower-level SIH (RBG 140–199 mg/dL) and higher-level SIH (RBG > 200 mg/dL) received a median of 4 cycles of chemotherapy; however, the reason for this is not discussed. Additionally, women with T2D and stage III BC were slightly less likely to receive chemotherapy than women with BC without T2D (RR 0.93 [95% CI 0.89–0.97]) [42]. However, this difference lost significance when adjusted for comorbidities (aHR 1.03 [95% CI 0.93–1.13]) [42]. Transient hyperglycaemia and SIH may have a greater impact on individuals with previously good glycaemic control compared to individuals with pre-existing diabetes [39].

A large multicentre study [43] focused on women with preexisting diabetes and BC, identifying chemotherapy treatment disparities in these individuals. Women with BC and preexisting diabetes were significantly less likely to receive chemotherapy (OR 0.67; 95% CI 0.48–0.93) and less likely to complete chemotherapy (OR 0.71; 95% CI 0.50–0.99) compared with women without diabetes. Of those who received guideline-recommended chemotherapy regimens, 69.7% of women with diabetes completed treatment compared to 76.3% of women without diabetes. These findings underscore the broader impact of diabetes on the delivery of chemotherapy in women with BC, highlighting the unmet need of women with BC and diabetes.

## Long-term hyperglycaemia and/or T2D in women with BC who have received cytotoxic chemotherapy

### Pathological response

There is a critical relationship between T2D, fasting plasma glucose (FPG) levels, and the pathological response in women with BC undergoing neoadjuvant chemotherapy [41]. Women with BC and T2D had a significantly lower pathological response rate than women with BC without T2D (*P* = 0.005), and elevated FPG levels were independently associated with poorer pathological response (*P* = 0.008). A cut-off FPG value of 105 mg/dL identified women at risk of a non-pathological response (sensitivity 85.7%, specificity 74.2%), suggesting a potential link between glucose control and treatment effectiveness.

Supporting these findings, a large Pakistani cohort (*n* = 1,372) [45] reported that women with T2D had significantly lower rates of pathological complete response than women without diabetes (3.9% vs. 14.9%, *p* < 0.001). Importantly, this study stratified responses beyond the traditional binary approach, showing women with diabetes also achieved lower pathological partial response rates (11.0% vs. 38.0%). Using multinomial logistic regression, non-diabetic status was associated with increased odds of achieving a pathological complete response (OR: 1.91, 95% CI: 1.26–2.89) and a pathological partial response (OR: 1.71, 95% CI: 1.24–2.37) compared to a pathological no response. This analysis provides crucial insights into how diabetes affects the entire spectrum of treatment response.

### Prognosis

Hyperglycaemia is an independent prognostic factor for poor relapse-free survival (RFS) but not overall survival (OS) [40]; 423 women without diabetes with BC were divided into two groups: euglycaemia and hyperglycaemia. The serum fasting glucose level, or RBG, was obtained once in the morning when individuals attended the hospital for chemotherapy. Therefore, serial measurements of blood glucose levels were not performed, as is typically done in patients with T2D. Hyperglycaemia occurred in 82 of 423 women, with 10 participants developing T2D post-chemotherapy. Propensity score matching (PSM) was conducted to balance patient characteristics between the euglycaemia and hyperglycaemia groups, resulting in 75 participants per group. Prior to PSM, significant differences existed between groups in participant characteristics (*p* = 0.039) and BMI (*p* = 0.001); these differences were resolved following matching. The proportion of women with hypertension (HTN) was (*n* = 14) in the hyperglycaemia group compared to (*n* = 7) in the euglycaemia group.

Additionally, in the hyperglycaemic group, 58 women had a BMI > 23, whereas in the euglycaemic group, 46 women had a BMI ≥ 23. The 5-year RFS rates were 92.0% and 82.3% in the euglycaemia and hyperglycaemia groups, respectively. Cancer recurrence occurred in (*n* = 7) women in the euglycaemia group and (*n* = 17) women in the hyperglycaemia group (*p* = 0.011). Four women in the euglycaemia group and nine in the hyperglycaemia group died, resulting in 5-year OS rates of 94.6% and 92.0%, respectively (*p* = 0.113).

Hyperglycaemia was identified as an independent prognostic factor for worse RFS (HR 3.504; 95% CI 1.390–8.836; *p* = 0.008) but not OS; the reason for this is not discussed, further reducing the validity of the findings.

### Mortality

A study of women undergoing chemotherapy for BC revealed that individuals with T2D faced increased all-cause mortality, even after accounting for comorbidities (aHR 1.16 [1.06–1.27]) [38]. Women with a T2D duration exceeding five years and preexisting cardiovascular disease were associated with elevated all-cause and BC-specific mortality rates. Initially, women with T2D presented significantly higher all-cause and BC-specific mortality rates than women with BC without T2D retrospectively (HR 1.42 and 1.24, respectively). These results remained consistent even after adjusting for chemotherapy.

## Discussion

This systematic review of nine studies [37–45] encompassing 26,251 women demonstrates that hyperglycaemia and T2D significantly compromise BC outcomes in women receiving cytotoxic chemotherapy. The evidence reveals a concerning cascade in which poor glycaemic control leads to both immediate treatment complications and long-term impact on survival rates.

Hyperglycaemia compromises optimal cancer treatment delivery. The co-occurrence of diabetes and cancer presents significant challenges [46, 47], with elevated healthcare utilisation, treatment-related toxicities, and modifications that impact therapeutic effectiveness. Whilst cancer management has evolved toward multidisciplinary team approaches [48], discrepancies in care provision for individuals with both conditions persist, creating avoidable complications.

Multiple mechanisms drive these complications. Glucocorticoids such as dexamethasone cause dose-dependent hyperglycaemia, by reducing insulin sensitivity, increasing hepatic gluconeogenesis, and impairing beta cell function [49]. Approximately 32% of steroid-treated individuals experience SIH, with glucose peaks often undetected at home due to the postprandial and transient nature of glucose elevations [19, 39]. Chemotherapy regimens combined with steroids necessitate dose reduction or interruption [37, 43], with incidence rates of hyperglycaemia during chemotherapy ranging from 19 to 67% depending on treatment protocol and threshold definition [39, 43]. Targeted therapies, including tyrosine kinase inhibitors and mTOR inhibitors, cause hyperglycaemia through distinct endocrine mechanisms [50], whilst immune checkpoint inhibitors present an emerging risk of insulin-dependent diabetes, including acute metabolic emergencies such as diabetic ketoacidosis [51–54], with clinical management guidance now provided in international guidelines [53]. This breadth of mechanisms highlights that glycaemic risk extends well beyond traditional cytotoxic chemotherapy [47, 50].

The consequences are substantial. Cancer-related mortality is 18% higher across all cancer types in individuals with diabetes, 9% higher for BC specifically, and the risk of colorectal cancer mortality is 2.4 times greater in individuals with T2D compared to the general population [55]. The impaired pathological responses, worse relapse-free survival, and elevated mortality documented across studies demonstrate that hyperglycaemia's impact extends far beyond the treatment period. Diabetes-related comorbidities may influence treatment choices, with individuals potentially receiving less aggressive treatments compared to individuals without diabetes, resulting in suboptimal outcomes [44, 55, 56].

### Fragmented care

The fragmented care coordination evident in these studies, particularly inadequate primary care follow-up for new-onset T2D after cancer diagnosis, creates critical care gaps [16, 56, 57]. Pinheiro et al. [16] documented that oncologists reported 'never doing diabetes management' whilst PCPs lacked knowledge of 'rapidly evolving cancer treatments.' Without routine updates between providers or integrated care teams, patients navigated complex medication interactions and SIH alone [16, 56]. This systemic fragmentation, compounded by patients and caregivers focusing on cancer treatment, likely contributes to the treatment modifications and inferior outcomes observed across studies in this review.

The solution requires integrated multidisciplinary teams [16, 48, 56, 57]. The emergence of cardio-oncology and onco-nephrology [58] provides precedent for 'diabeto-oncology' as a subspecialty. Clinicians with training covering chemotherapy-induced hyperglycaemia, steroid protocols, metabolic emergencies, and long-term metabolic effects [16, 48] could provide the coordinated expertise needed to prevent treatment disruptions and optimise outcomes for this vulnerable population.

### Screening/Education

Approximately 70–80% of women diagnosed with BC receive curative-intent treatment [7], yet 20% of individuals with cancer have co-occurring T2D at the time of cancer diagnosis [16]. Given this review's findings that hyperglycaemia and T2D significantly compromise treatment delivery and outcomes, systematic pre-chemotherapy screening is essential, particularly for women with risk factors including high BMI, physical inactivity, family history, or gestational diabetes [19].

Baseline screening should identify individuals requiring close glucose monitoring throughout chemotherapy [19]. For those at increased risk, recommendations include fasting plasma glucose monitoring every 2 weeks during the first month, followed by monthly monitoring, with HbA1c testing at baseline, 3 months, and annually [19]. However, given that frequent anaemia and blood transfusions in this population render HbA1c unreliable [19], self-monitoring of blood glucose may be preferable during active treatment [19].

Effective self-management becomes critical when hyperglycaemia compromises treatment completion [59]. Structured educational interventions should address glucose fluctuations during chemotherapy, recognition and management of hyperglycaemia, and practical skills such as self-monitoring, dietary modifications, infection control, and stress management [59]. Person-centred approaches should incorporate collaborative goal setting aligned with treatment milestones, such as stabilising glucose during initial cycles to prevent dose reductions [60]. Technology-facilitated monitoring through mobile applications and continuous glucose monitoring [60] may help women navigate glycaemic challenges, whilst psychological support addressing the emotional burden of concurrent disease management is essential [59, 60].

Management requires individualised care balancing glycaemic control with treatment tolerability and quality of life [61]. Targets must be tailored to performance status, life expectancy, disease stage, hypoglycaemic risk, comorbidities, and available support [61]. For women receiving curative-intent treatment, targets should optimise treatment completion whilst preventing long-term complications [61]. However, absent randomised trials demonstrating that tight control improves cancer outcomes, rigid adherence to stringent targets may impose unnecessary burden [47, 61]. The primary goal remains preventing severe hyperglycaemia that necessitates treatment modifications whilst avoiding hypoglycaemia [61].

Healthcare providers must recognise that managing concurrent diabetes and cancer affects individuals differently — some find diabetes management provides control during cancer treatment, whilst others find it overwhelming [61]. A compassionate approach requires flexibility to liberalise targets when this reduces distress [61]. For individuals with advanced disease, targets should prioritise comfort and quality of life, prevent symptomatic hyperglycaemia whilst minimising intrusive monitoring [19, 47, 61]. As individuals transition through treatment, glycaemic management requires ongoing reassessment in partnership with the individual, adjusting goals to reflect changing priorities and circumstances [61].

Oncology nurses play crucial roles in implementing diabetes-oncology care integration through systematic screening, patient education, and collaboration with diabetes specialist teams [59, 62]. Their multifaceted role encompasses thorough assessments, symptom identification, and development of individualised care plans [61, 62]. Through effective communication, nurses foster supportive environments that empower individuals to take ownership of their care, providing critical information on medication adherence, glucose monitoring, follow-up care, and complication management [16, 62]. This holistic approach addresses the physical, emotional, social, and educational needs of individuals and families [61, 62].

### Future research

While this review demonstrates consistent associations between diabetes/hyperglycaemia and adverse outcomes, critical gaps in evidence remain regarding optimal glycaemic management strategies. Prospective studies are urgently needed to determine whether intensive glucose control improves cancer-specific outcomes. Future research should adopt stratified response frameworks [45] and incorporate serial glucose monitoring to establish optimal treatment thresholds for individual patients [63]. Additionally, management strategies for hyperglycaemia induced by newer targeted therapies require investigation, as current recommendations are based on general diabetes principles rather than cancer-specific evidence [47, 63].

### Limitations

The restriction to BC studies limits broader applicability, although this focus was necessary due to limited evidence on other tumour sites and ensured sufficient homogeneity for synthesis. The retrospective nature of all nine studies introduces selection bias and incomplete data capture. Variable definitions of diabetes across studies, differing classification approaches for pathological response, and limited monitoring data (HbA1c, serial glucose measurements) compromise the consistency and comparability of findings. North American healthcare systems are overrepresented, which limits the direct transferability of findings to European and UK clinical practice.

## Conclusion

The identification and management of hyperglycaemia and/or T2D is crucial to reduce complications and improve outcomes in women with BC who are undergoing or have completed cytotoxic chemotherapy. While published guidance is available [19], embedding this information in clinical practice requires an MDT approach [48]. The development of comprehensive glycaemic management strategies and the provision of personalised care are vital to avoid complications and, thereby, inferior outcomes in women with BC. However, the absence of robust evidence demonstrating that intensive glucose control improves cancer-specific outcomes [47] requires individualised approaches that balance potential benefits against risks of aggressive management during the physiological stress of cancer treatment. Providing this level of care has the potential to prevent short- and long-term consequences of treatment, thereby improving the quality of life with and beyond a diagnosis of BC.

## Supplementary Information

Below is the link to the electronic supplementary material.

ESM 1 Supplementary Material 1 (PDF 11.6 KB)

ESM 2 Supplementary Material 2 (PDF 11.4 KB)

ESM 3 Supplementary Material 3 (PDF 14.1 KB)

ESM 4 Supplementary Material 4 (PDF 11.3 KB)

## Data Availability

Data sharing is not applicable to this article as no datasets were generated or analysed during the current study.

## Declarations

### Use of artificial intelligence

Artificial intelligence tools (Claude and Grammarly) were used to assist with language editing, grammar checking, and document formatting. No AI tools were used for literature searching, data extraction, quality appraisal, analysis, or interpretation of results. The lead author (A.C.) reviewed and verified all AI-assisted content to ensure accuracy, integrity, and alignment with the research findings. All intellectual content, including the conceptualisation, analysis, and conclusions, remains the sole responsibility of the authors.
