# Supplementary figures and images for: The impact of hyperglycaemia and/or type 2 diabetes on women with breast cancer undergoing or post-cytotoxic chemotherapy: a systematic literature review
Source: Support Care Cancer. 2026 Jun 30;34(7):709. doi: 10.1007/s00520-026-10947-w (PMC13319691; doi:10.1007/s00520-026-10947-w)

# Healthcare Utilisation

● Emergency Visits

● Hospital Admissions

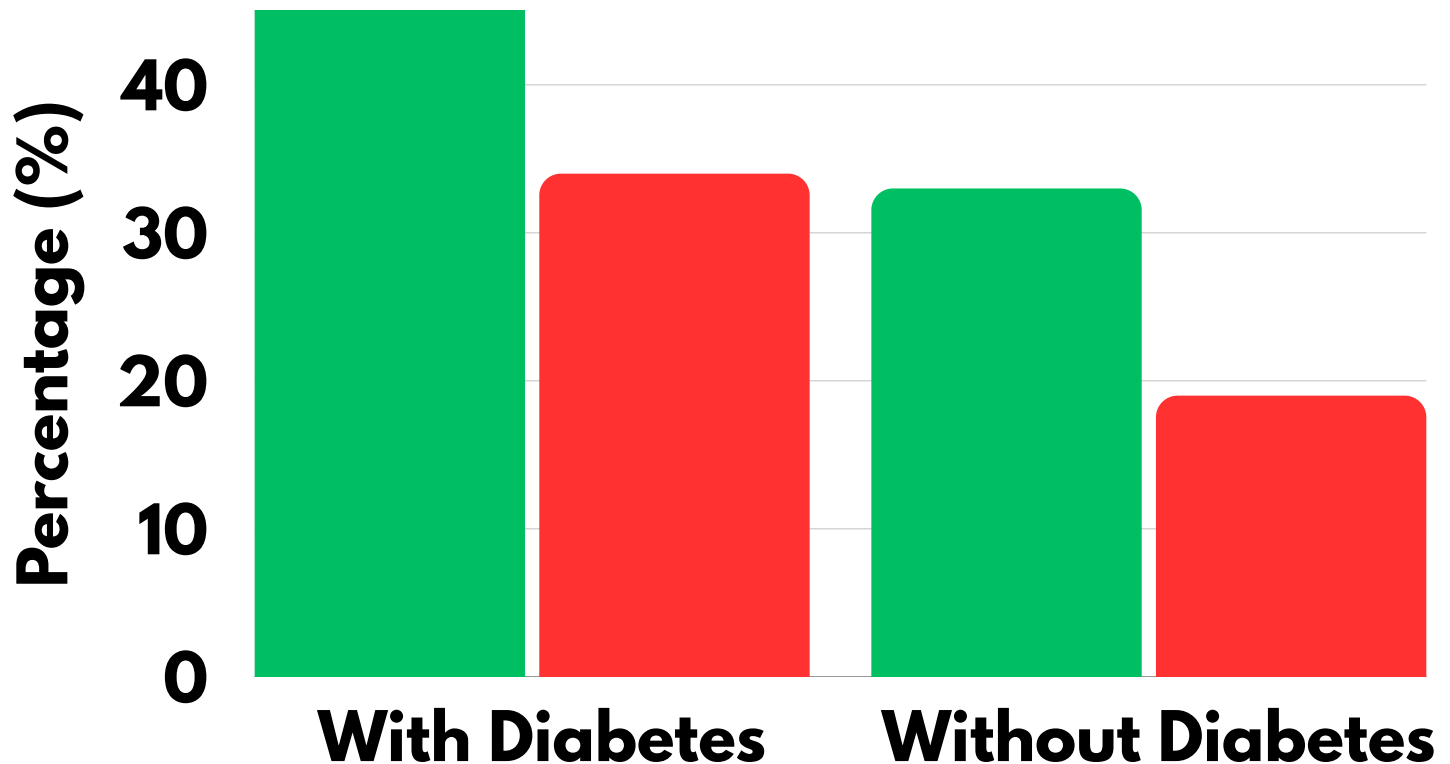

Supplement: Supplementary file 1 — Supplementary Material 1 (PDF 11.6 KB) [file 520_2026_10947_MOESM1_ESM.pdf]

# Pathological Response

● Complete Response

● Partial Response

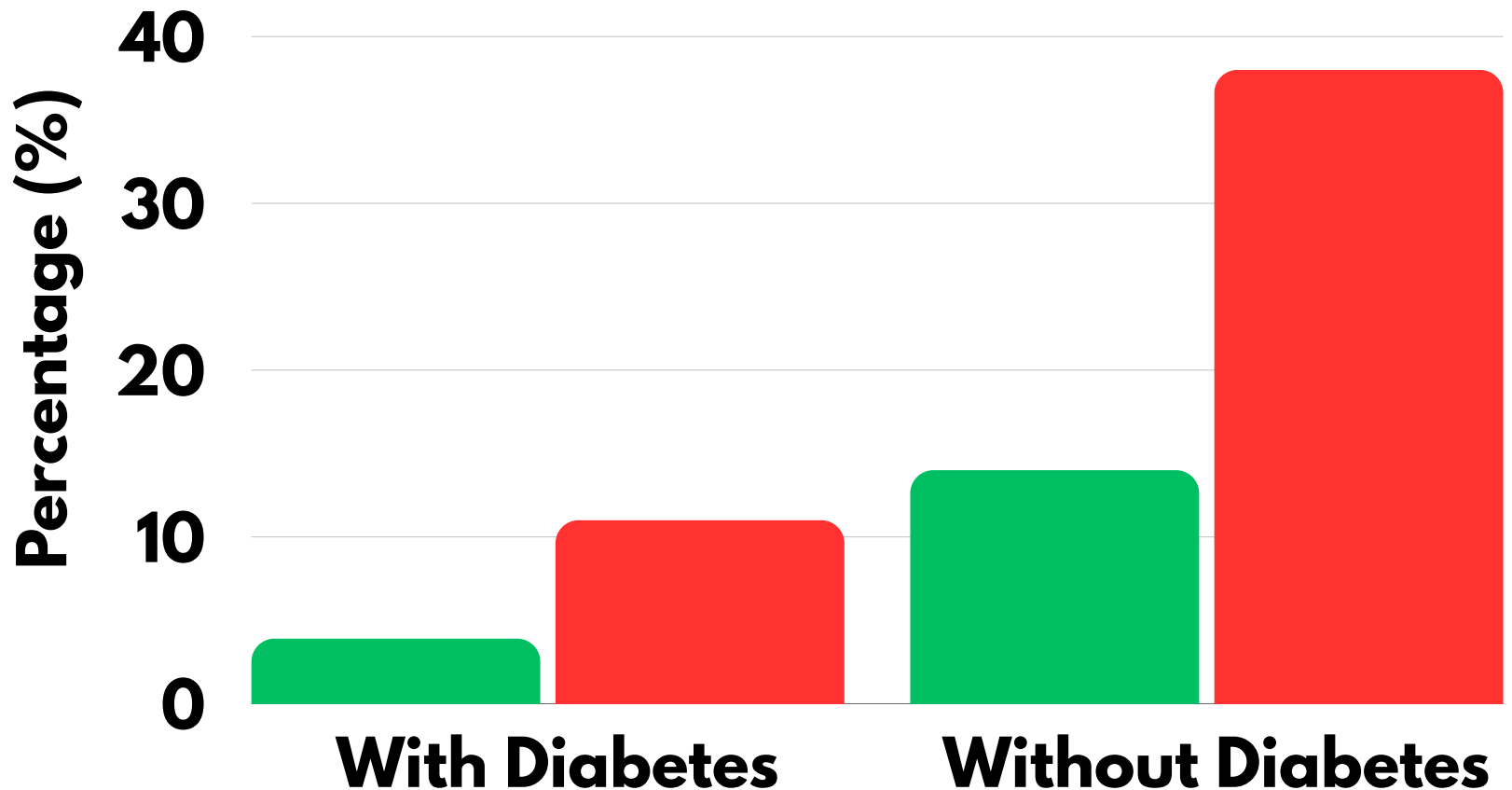

Supplement: Supplementary file 2 — Supplementary Material 2 (PDF 11.4 KB) [file 520_2026_10947_MOESM2_ESM.pdf]

# 5-Year Survival Comparison

**Relapse**

**18%**

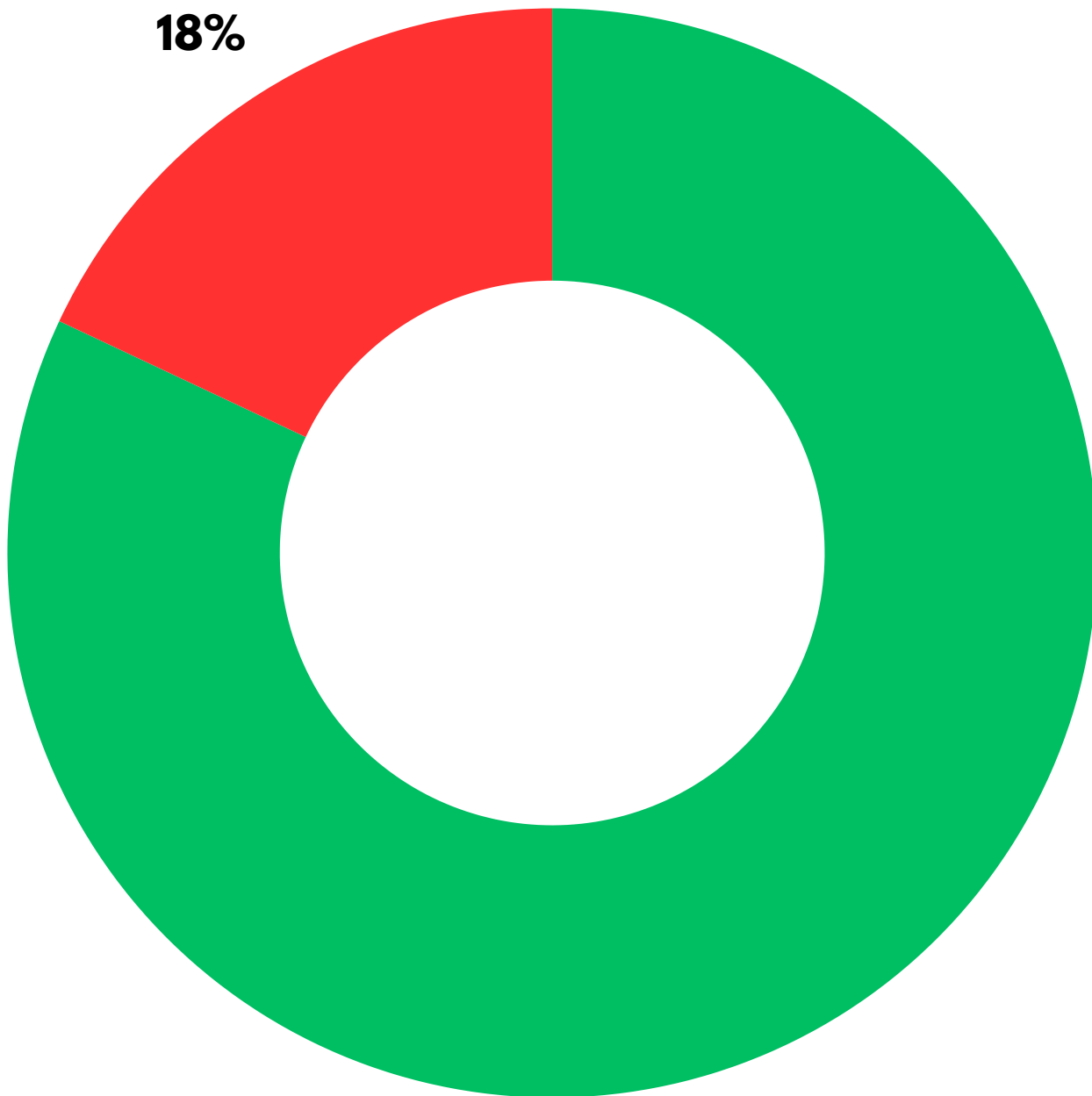

**5- Year Relapse- Free Survival**

**82%**

Supplement: Supplementary file 3 — Supplementary Material 3 (PDF 14.1 KB) [file 520_2026_10947_MOESM3_ESM.pdf]

# Treatment Modification

● Dose Omission

● Incomplete Regimens

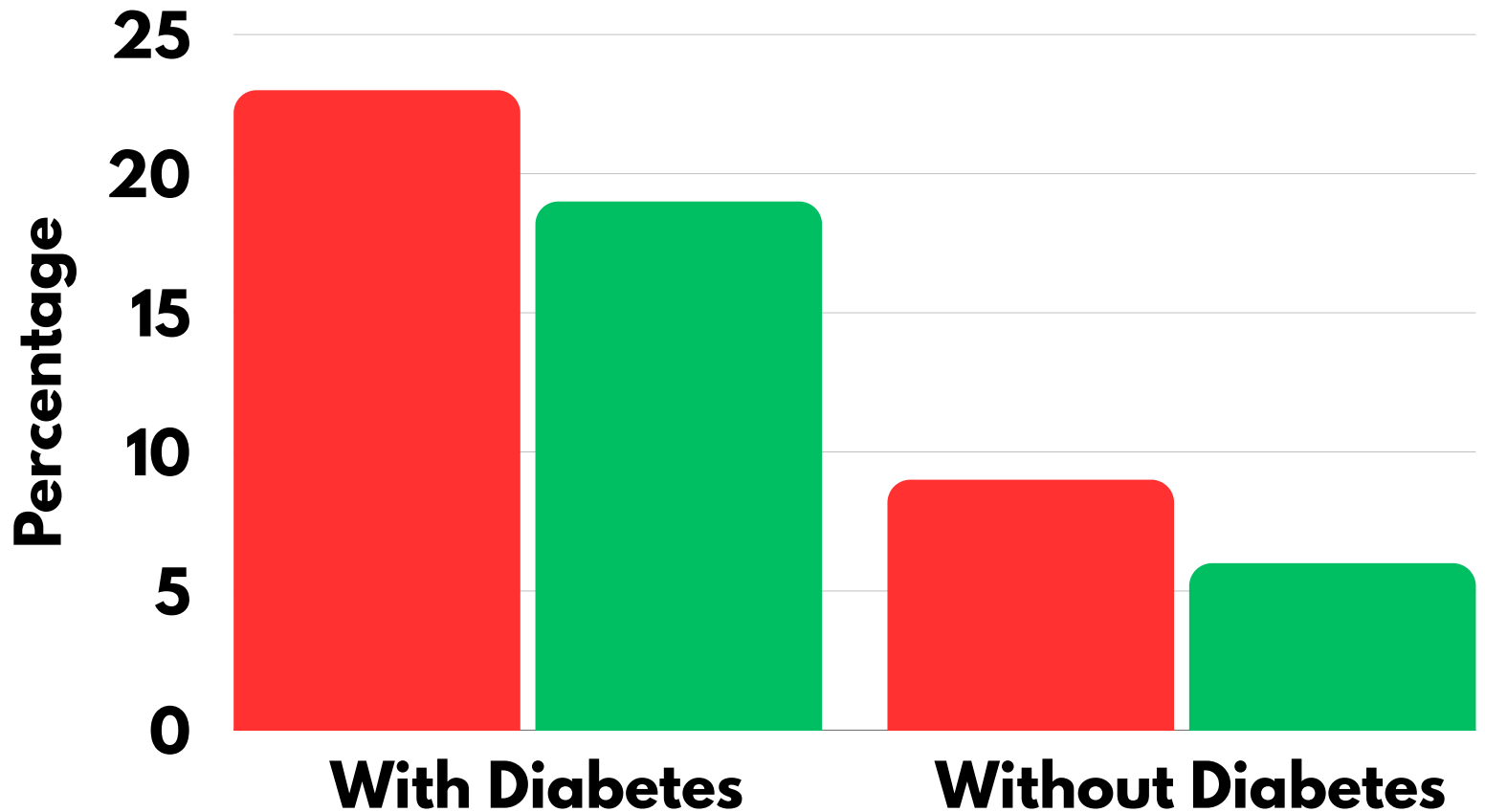

Supplement: Supplementary file 4 — Supplementary Material 4 (PDF 11.3 KB) [file 520_2026_10947_MOESM4_ESM.pdf]
